# The Resistance Mechanism of *Mycoplasma bovis* From Yaks in Tibet to Fluoroquinolones and Aminoglycosides

**DOI:** 10.3389/fvets.2022.840981

**Published:** 2022-08-02

**Authors:** Jiaqiang Niu, Mingshuai Yan, Jinhua Xu, Yefen Xu, Zhenyu Chang, Suolang Sizhu

**Affiliations:** Tibet Agriculture and Animal Husbandry College, Linzhi, China

**Keywords:** fluoroquinolones, resistance mechanism, yak, *Mycoplasma bovis*, aminoglycosides

## Abstract

*Mycoplasma bovis* (*M. bovis*) is one of the important pathogens for yaks. Aminoglycosides and fluoroquinolones are frequently used medications for the treatment of *M. bovis*. Drug-resistant strains were inevitable with the abuse of antibiotics. The resistance of *M. bovis* to aminoglycosides was related to the base mutations in drug target genes. Amino acid mutations at the quinolone resistance-determining region (QRDR) in gyrA, gyrB, parC, and parE conferred resistance to fluoroquinolones. In order to investigate the resistance mechanism of *M. bovis* from yaks in Tibet to aminoglycosides and fluoroquinolones, six frequently used antibiotics and ten clinical *M. bovis* strains were administered for a drug sensitivity test for *in vitro*-induced highly resistant strains, a drug stable-resistance test, cross-resistance test, and analysis of target gene mutations. The results showed that the clinical strains of *M. bovis* from yaks in Tibet had varying degrees of resistance to fluoroquinolones and aminoglycosides. The mechanism of resistance to fluoroquinolones and aminoglycosides was identified preliminarily for *M. bovis* from yaks: the single-site base mutation mediated the resistance of *M. bovis* from yaks and both base mutations led to highly resistant strains (aminoglycosides: rrs3 and rrs4; fluoroquinolones: gyrA and parC). The active efflux system results of M. bovis showed that there was no active efflux system based on fluoroquinolones and aminoglycosides expressed in *M. bovis* from yaks. The research could provide a reference for clinical treatment of *M. bovis*.

## Introduction

*Mycoplasma bovis* (*M. bovis*) is one of the important pathogens that causes bovine disease syndromes such as pneumonia, mastitis, keratoconjunctivitis, arthritis, genital tract inflammation, miscarriage, and infertility (1). Sick cattle and the respiratory modes of transmission were the main sources and routes of infection (2). *Mycoplasma* was isolated firstly from mastitis milk by Hale et al. (3). It caused huge economic losses to the cattle industry (4).

In China, *M. bovis* was first isolated by Li (5) and proved to be the pathogen of respiratory diseases in cattle until 2008 (5–8). Antibiotics (aminoglycosides and fluoroquinolones) have been used to treat *M. bovis*. Previous research confirmed the resistance to macrolides and fluoroquinolones for *M. bovis* clinically isolated strains (9, 10). In our previous study, we found that the isolated strains were resistant to macrolides, aminoglycosides, and lincosamides (11). The research was imperative for drug resistance mechanisms (12).

Aminoglycosides and fluoroquinolones are broad-spectrum antibiotics against Gram-negative bacteria (13). The resistance of *M. bovis* to aminoglycosides is related to the base mutations in drug target genes (14). Previous research showed that there was a base mutation in 16S rRNA, but no base mutation was detected in S12 ribosomal protein (15–17). Amino acid mutations at the quinolone resistance-determining region (QRDR) in gyrA, gyrB, parC, and parE conferred resistance to fluoroquinolones (18). The mutation in the QRDR of gyrA contributed to nalidixic acid resistance (19). The amino acid mutation in parC was the leading cause of fluoroquinolones resistance in bacteria (20). The efflux pump mediated the resistance to aminoglycosides by AmrB, MexY, and AcrD genes and to fluoroquinolones by EmrAB_‘_ AcrAB_‘_ YdhE_‘_ AcrEF, and MdfA genes in resistance nodulation cell division (21, 22).

In our study, we performed antibiotic susceptibility testing, drug target gene mutation analysis, and developed a preliminary confirmation of an active drug efflux system on 10 isolates of *M. bovis* from Tibet yaks. It could provide references for the drug-resistant mechanism of *M. bovis*.

## Materials and Methods

### Strains

Ten isolated strains of *M. bovis* from yaks in Tibet (Tibet-1~10) came from the Key Laboratory of Tibet Plateau Animal Disease Research Autonomous Region (11).

### Design and Synthesis of Primers

The DNA of *M. bovis* was extracted by the boiling method (23). The primers were designed as described in a previous research paper (Table 1) (17, 18).

**Table 1:** Primer sequences.

**Gene name**	**Primer sequence (5′ → 3′)**	**Tm (**°**C)**	**Product length**
rrs 3	F: GGATATCTAACGCCGTGTC	50°C	1,857 bp
	R: CGTTCTCGTAGGGATACCT		
rrs 4	F: GAGTTTGATCCTGGCTC	43°C	1,812 bp
	R: GTATTTTCCTATTGTTGTTA		
rps E	F: GCATGGCAGATTTAGAAAACAAGA	51°C	696 bp
	R: CGGTGCTTAACCTAAAAGGTCTTTA		
gyr A	F:GACGAATCATCTAGCGAG	56°C	531 bp
	R:GCCTTCTAGCATCAAAGTAGC		
gyr B	F:CCTTGTTGCCATTGTGTC	56°C	555 bp
	R:CCATCGACATCAGCATCAGTC		
par C	F:GGTACTCCTGAAGCTAAAAGTGC	56°C	488 bp
	R:GAATATGTGCGCCATCAG		
par E	F:GAGCAACAGTTAAACGATTTG	56°C	502 bp
	R:GGCATAACAACTGGCTCTT		

*PCR, reaction conditions: 95°C pre-denaturation 3 min, (95°C denaturation 30 s, Tm °C annealing 30 s, 72°C extension 45 s, 30 cycles), extension 10 min at 72°C*.

### Antibiotic Susceptibility Testing

The minimum inhibitory concentration (MIC) of the 10 strains were detected by the microdilution method (17) against SPE (spectinomycin), GEN (gentamicin), KAN (kanamycin), ENR (enrofloxacin), NOR (norfloxacin), and CIP (ciprofloxacin). The drug resistance results referred to the CLSI (Clinical and Laboratory Standards Institute, USA) standards (SPE: MIC≥128 μg/mL; GEN,KAN: MIC≥32 μg/mL; ENR,NOR,CIP: MIC≥4 μg/mL) (24).

### Cross-Resistance Test

The highly resistant strains of *M. bovis in vitro* were induced until there was 512 μg/mL of SPE, GEN, KAN, NOR, CIP, and 256 μg/mL of ENR. There were only three highly resistant strains induced successfully (Tibet-1, Tibet-6, and Tibet-8, and named *M. bovis* 1, *M. bovis* 6, and *M. bovis* 8). The highly resistant strains to ENR, NOR, and CIP were tested for cross-resistance for the highly resistant stains to SPE, GEN, and KAN.

### Antibiotic Target Mutation Analysis

The DNA was extracted by water-boiling and amplified by PCR for the sensitive, resistant, and *in vitro*-induced strains. The PCR products were recovered using the Gel Extraction Kit (Omega Bio-Tek Co., Ltd., USA) and connected to the pMD18-T. The recombinant plasmid was verified by M13 and sequenced by Shenggong Bioengineering (Shanghai) Co., Ltd. DNAMAN software was used to compare and analyze the sequencing results.

### Overexpression of the Active Efflux System of *M. bovis*

The MIC of CCCP (carbonyl cyanide m-chlorophenyl hydrazone) and VP (verapamil) was detected for the sensitive, resistant, and *in vitro*-induced strains. The overexpression of the active efflux system was judged to exist when the MIC of antibiotics using CCCP and VP was less than 1/4 of the original MIC value.

## Results

### Antibiotic Susceptibility Testing

The MIC results of the 10 strains showed that Tibet-1 was resistant to CIP; Tibet-6 was resistant to SPE, GEN, CIP, and ENR; Tibet-7 was resistant to NOR; Tibet-8 was resistant to SPE and CIP; Tibet-9 was resistant to NOR; and other isolates were relatively sensitive to these antibiotics (Table 2).

**Table 2:** The MIC results of the 10 strains (ug/mL).

**Strain name**	**MIC (ug/mL)**					
	**SPE**	**GEN**	**KAN**	**CIP**	**ENR**	**NOR**
Tibet-1	64	16	8	8	2	1
Tibet-2	32	8	8	2	2	0.25
Tibet-3	32	8	4	2	1	0.25
Tibet-4	16	2	2	1	0.5	2
Tibet-5	16	2	16	0.5	2	0.5
Tibet-6	128	64	2	4	8	2
Tibet-7	8	8	2	0.5	0.25	4
Tibet-8	128	1	4	4	0.5	1
Tibet-9	4	1	1	1	0.25	4
Tibet-10	4	2	8	0.5	1	0.5

*SPE, spectinomycin; GEN, gentamicin; KAN, kanamycin; CIP, ciprofloxacin; ENR, enrofloxacin; NOR, norfloxacin*.

### Detection of Cross-Resistance

Three resistant strains to one fluoroquinolone antibiotic were used for cross-resistance to the other fluoroquinolone antibiotics and the same for aminoglycosides. The results showed that there was cross-resistance (Table 3).

**Table 3:** Test results of cross-resistance induced *in vitro* (ug/mL).

**Strain name**	**Inducing drug**	**MIC (ug/mL)**			**Inducing drug**	**MIC (ug/mL)**		
	**concentration μg/mL**	**CIP**	**ENR**	**NOR**	**concentration μg/mL**	**SPE**	**GEN**	**KAN**
*M. bovis* 1	CIP (512)	512	128	128	SPE (512)	512	128	128
	ENR (256)	128	256	64	GEN (512)	128	512	64
	NOR (512)	64	64	512	KAN (512)	128	64	512
*M. bovis* 6	CIP (512)	512	64	256	SPE (512)	512	256	64
	ENR (256)	256	256	256	GEN (512)	128	512	32
	NOR (512)	128	32	512	KAN (512)	256	256	512
*M. bovis* 8	CIP (512)	512	256	256	SPE (512)	512	256	256
	ENR (256)	32	256	128	GEN (512)	128	512	64
	NOR (512)	128	64	512	KAN (512)	128	128	512

*SPE, spectinomycin; GEN, gentamicin; KAN, kanamycin; CIP, ciprofloxacin; ENR, enrofloxacin; NOR, norfloxacin. M. bovis 1, M. bovis 6, M. bovis 8: the highly resistant strains induced in vitro*.

### PCR Results of Target Genes

**Figure 1:**
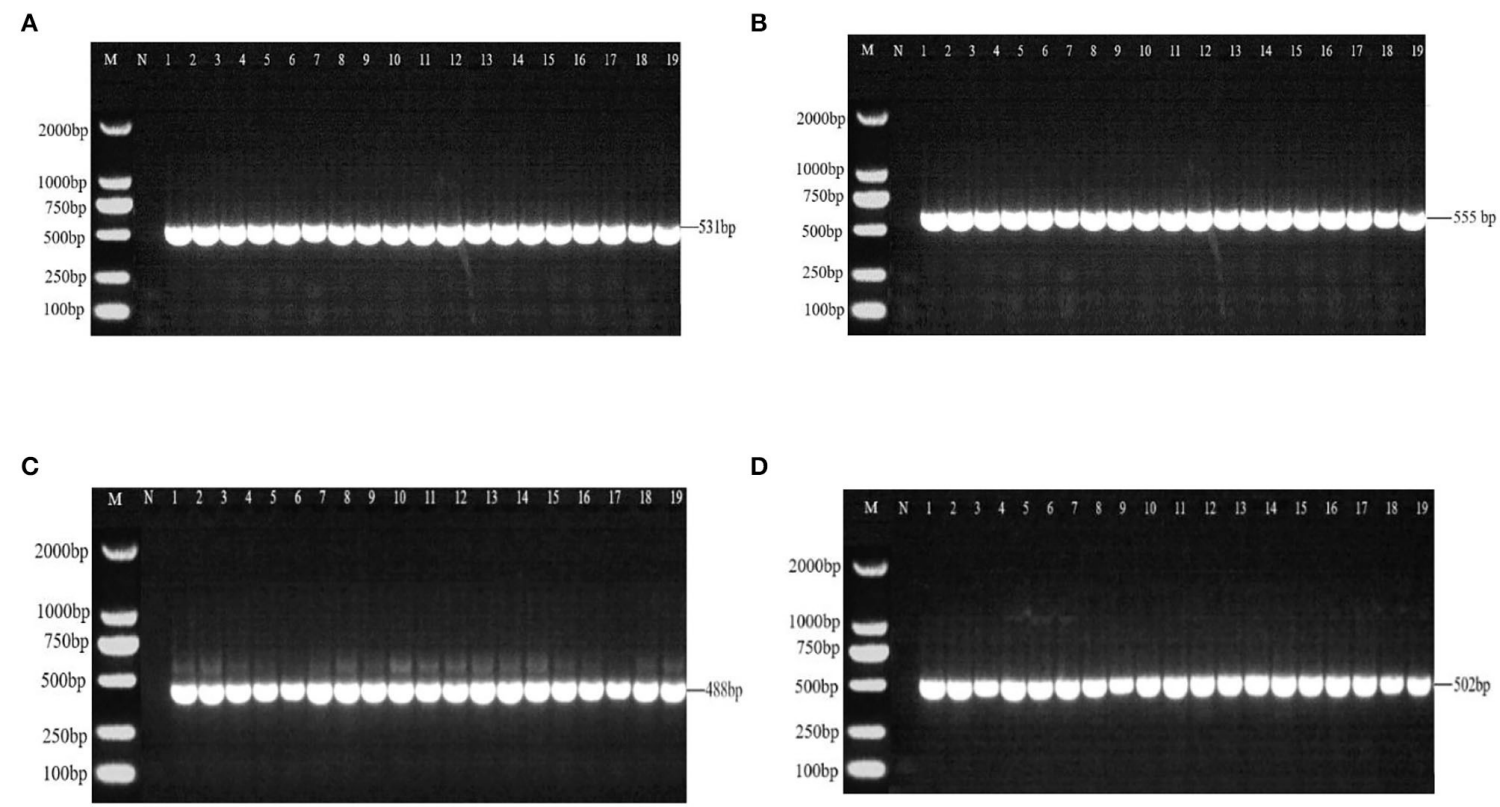
PCR results of drug-resistant target genes gyrA **(A)**, gyr B **(B)**, parC **(C)**, and parE **(D)**. M: DL2000; N: Negative control; 1~10: Tibet 1-10; 11~13: CIP-inducible strains; 14~16: ENR-inducible strains; 17 ~19: NOR-inducible strains.

**Figure 2:**
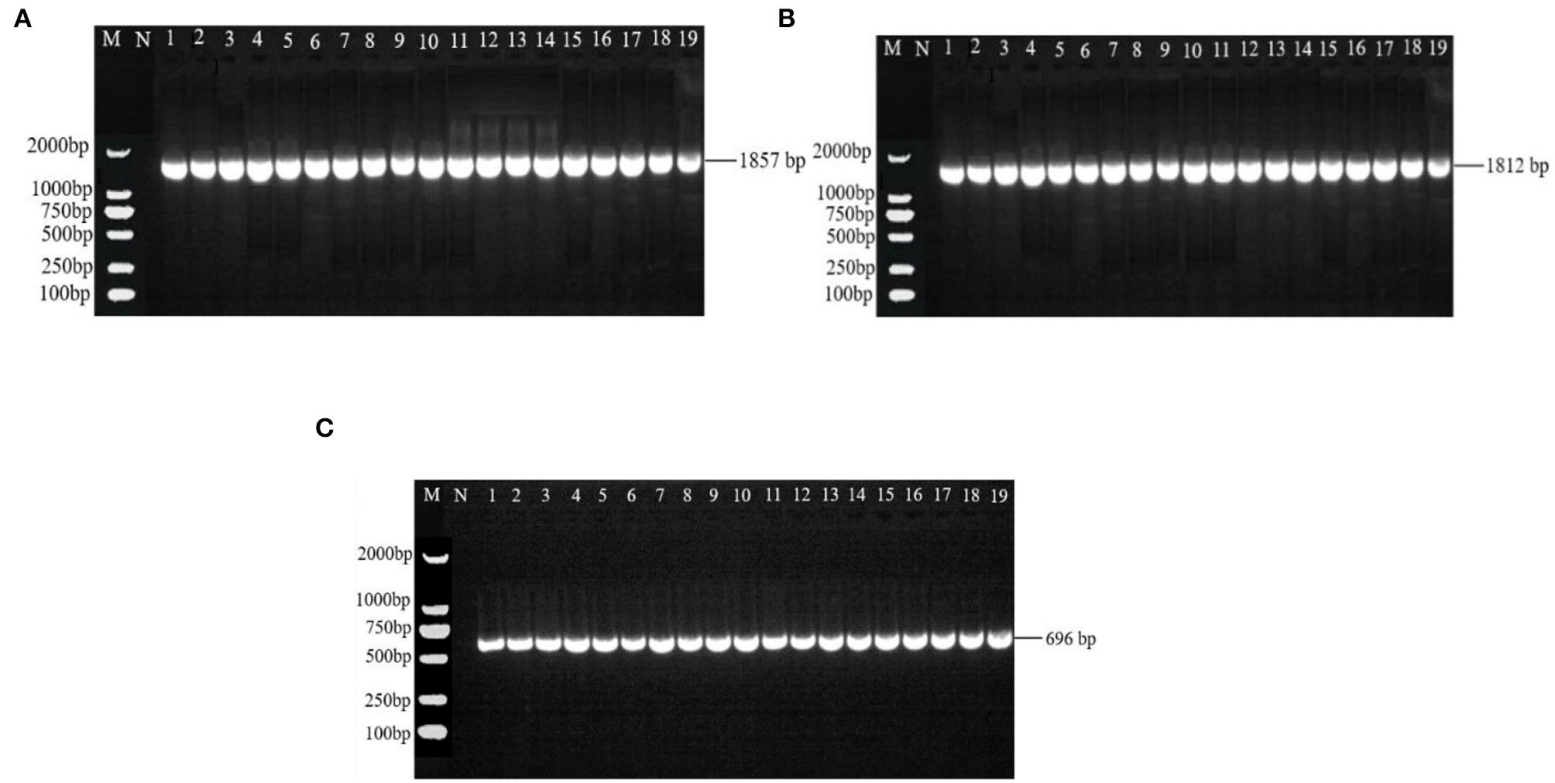
PCR results of drug-resistant target genes rrs3 **(A)**, rrs4 **(B)**, and rpsE **(C)**. M: DL2000; N: Negative control; 1~10: Tibet 1-10; 11~13: SPE-inducible strains; 14~16: GEN-inducible strains; 17~19: KAN-inducible strains.

There were 531 bp, 555 bp, 488 bp, and 502 bp fragments in gyrA, gyrB, parC, and parE genes for the susceptible strains, drug-resistant strains, and *in vitro*-induced strains to fluoroquinolones (Figure 1); there were 1,857, 1,812, and 696 bp fragments in rrs3, rrs4, and rpsE genes for the susceptible strains, drug-resistant strains, and *in vitro*-induced strains to aminoglycosides (Figure 2).

### Analysis of Target Gene Mutations

The mutation analysis of gyrA, gyrB, parC, and parE showed that there was a nonsense mutation in parC (GAC84GAT) of clinically sensitive strains. Mutated amino acids Ser83Phe or Tyr, Ser80Ile or Arg, or Ser81Phe were detected due to base mutations TCT83TTT or TAT, AGC80ATT or AGA, or TCT81TTT in gyrA or parC of six clinically resistant strains (Table 4). There were multiple base mutations in gyrA and parC of nine strains induced *in vitro*, the strains were highly resistant to fluoroquinolones: the amino acids Gly81Cys, Ser83Phe, and Glu87Asp or Lys were detected to be mutated due to the base mutations of GGT81TGT?TCT83TTT, and GAA87GAT orAAA in gyrA; the amino acids Ser80Ile, Ser81Tyr, and Asp84Asn or Tyr were detected to be mutated with the base mutations of AGT80ATT, TCT81TAT, and GAC84AAT or TAT in parC (Table 5).

**Table 4:** The QRDR mutations of clinical drug-resistant strains.

**Strain name**	**Gene mutation site situation**				
	**gyrA**	**gyrB**	**parC**		**parE**

	**Ser83(TCT)**		**Ser 80 (AGT)**	**Ser 81 (TCT)**	
Tibet-1 CIP	Phe (TTT)	-	-	-	-
Tibet-6 CIP	-	-	Ile (ATT)	-	-
Tibet-8 CIP	Phe (TTT)	-	-	-	-
Tibet-6 ENR	Tyr (TAT)	-	-	-	-
Tibet-7 NOR	-	-	-	Phe (TTT)	-
Tibet-9 NOR	-	-	Arg (AGA)	-	-

*Tibet-1 CIP, The clinical drug-resistant strain to ciprofloxacin; the same for Tibet-6 CIP; Tibet-8 CIP; Tibet-6 ENR; Tibet-7 NOR; Tibet-9 NOR*.

**Table 5:** The QRDR mutations of strains induced *in vitro* to fluoroquinolones.

**Strain name**	**Gene mutation site situation**							
	**gyrA**			**gyrB**	**parC**			**parE**
	**Gly 81 (GGT)**	**Ser 83 (TCT)**	**Glu 87 (GAA)**		**Ser 80 (AGT)**	**Ser 81 (TCT)**	**Asp84 (GAC)**	
*M. bovis* 1 CIP		Phe (TTT)		-	Ile (ATT)			-
*M. bovis* 1 ENR		Phe (TTT)		-		Tyr (TAT)		-
*M. bovis* 1 NOR		Phe (TTT)		-	Ile (ATT)			-
*M. bovis* 6 CIP	Cys (TGT)		Asp (GAT)	-	Ile (ATT)			-
*M. bovis* 6 ENR		Phe (TTT)		-	Ile (ATT)			-
*M. bovis* 6 NOR		Phe (TTT)		-		Tyr (TAT)		-
*M. bovis* 8 CIP			Lys (AAA)	-	Ile (ATT)		Asn (AAT)	-
*M. bovis* 8 ENR			Lys (AAA)	-		Tyr (TAT)		-
*M. bovis* 8 NOR		Phe (TTT)		-			Tyr (TAT)	-

*M. bovis 1 CIP, The high drug-resistant strains induced in vitro to ciprofloxacin; the same for the others*.

The mutation analysis of rrs3, rrs4, and rpsE showed that there were no base mutation in clinically sensitive and resistant strains. There were base mutations in rrs3 and rrs4 of nine strains induced *in vitro*: it was a base mutation of A1409T in rrs3 and rrs4 of the highly resistant strain to GEN; there was a base mutation of A1408G in rrs3 and rrs4 of the highly resistant strain to KAN; one highly resistant strain to SPE had a C1192T base mutation in rrs3 and rrs4, and two strains had a C1192T base mutation in rrs3. Sense mutations were not detected in the rpsE-encoding ribosomal protein S5 (Table 6).

**Table 6:** The mutations of target genes in strains induced *in vitro* to aminoglycosides.

**Strain name**	**Gene mutation site situation**		
	**rrs 3**	**rrs 4**	**rps E (S5)**
*M. bovis* 1 GEN	A1409G	A1409G	-
*M. bovis* 1 KAN	A1408T	A1408T	-
*M. bovis* 1 SPE	C1192T	C1192T	-
*M. bovis* 6 GEN	A1409G	A1409G	-
*M. bovis* 6 KAN	A1408T	A1408T	-
*M. bovis* 6 SPE	C1192T	-	-
*M. bovis* 8 GEN	A1409G	A1409G	-
*M. bovis* 8 KAN	A1408T	A1408T	-
*M. bovis* 8 SPE	C1192T	-	-

*M. bovis 1 GEN, The high drug-resistant strains induced in vitro to gentamicin; the same for the others*.

### The Result of the Active Efflux System

The MIC of six antibiotics had no changes after using CCCP and VP, the result showed that there was no active efflux system based on fluoroquinolones and aminoglycosides expressed in *M. bovis* from Tibet yaks (Tables 7, 8).

**Table 7:** The MIC effects of fluoroquinolones by using CCCP and VP.

**Strain**	**MIC (ug/mL)**								
	**CIP**	**CCCP**	**VP**	**ENR**	**CCCP**	**VP**	**NOR**	**CCCP**	**VP**
	**CIP**				**ENR**	**ENR**		**NOR**	**NOR**
Tibet-1	8	8	8	2	2	2	1	1	1
Tibet-2	2	2	2	2	2	2	0.25	0.25	0.25
Tibet-3	2	2	2	1	1	1	0.25	0.25	0.25
Tibet-4	1	1	1	0.5	0.5	0.5	2	2	2
Tibet-5	0.5	0.5	0.5	2	2	2	0.5	0.5	0.5
Tibet-6	4	4	4	8	8	8	2	2	2
Tibet-7	0.5	0.5	0.5	0.25	0.25	0.25	4	4	4
Tibet-8	4	4	4	0.5	0.5	0.5	1	1	1
Tibet-9	1	1	1	0.25	0.25	0.25	4	4	4
Tibet-10	0.5	0.5	0.5	1	1	1	0.5	0.5	0.5
*M. bovis* 1	512	512	512	256	256	256	512	512	512
*M. bovis* 6	512	512	512	256	256	256	512	512	512
*M. bovis* 8	512	512	512	256	256	256	512	512	512

*Tibet 1-10: the clinical strains; M. bovis 1, 6, and 8: the highly resistant strains induced in vitro*.

**Table 8:** The MIC effects of aminoglycosides by using CCCP and VP.

**Strain**	**MIC (ug/mL)**								
	**SPE**	**CCCP**	**VP**	**GEN**	**CCCP**	**VP**	**KAN**	**CCCP**	**VP**
		**SPE**	**SPE**		**GEN**	**GEN**		**KAN**	**KAN**
Tibet-1	64	64	64	16	16	16	8	8	8
Tibet-2	32	32	32	8	8	8	8	8	8
Tibet-3	32	32	32	8	8	8	4	4	4
Tibet-4	16	16	16	2	2	2	2	2	2
Tibet-5	16	16	16	2	2	2	16	16	16
Tibet-6	128	128	128	64	64	64	2	2	2
Tibet-7	8	8	8	8	8	8	2	2	2
Tibet-8	128	128	128	1	1	1	4	4	4
Tibet-9	4	4	4	1	1	1	1	1	1
Tibet-10	4	4	4	2	2	2	8	8	8
*M. bovis* 1	512	512	512	512	512	512	512	512	512
*M. bovis* 6	512	512	512	512	512	512	512	512	512
*M. bovis* 8	512	512	512	512	512	512	512	512	512

*Tibet 1-10: the clinical strains; M. bovis 1, 6, and 8: the highly resistant strains induced in vitro*.

## Discussion

*M. bovis* is one of the most important pathogens that causes bovine respiratory syndrome (25). Therefore, *M. bovis* depends on drug treatment without a commercial vaccine outside the United States (4). The strain is relatively sensitive to aminoglycosides and fluoroquinolones. However, the large-scale use of antibiotics is contributing to the development of resistance. The action of the drug can be blunted due to drug resistance, thus affecting the health of animals and human (12).

In our study, the antibiotic susceptibility testing showed Tibet-1 was resistant to CIP; Tibet-6 was resistant to SPE, GEN, CIP, and ENR; Tibet-7 was resistant to NOR; Tibet-8 was resistant to SPE and CIP; and Tibet-9 was resistant to NOR. Half of the isolated strains were detected to be drug-resistant and Tibet-6 was resistant to four antibiotics. The drug resistance of *M. bovis* from yaks could not be ignored.

There was limited information about the resistance mechanism of *M. bovis* from yaks. In our research, the mutation analysis of rrs 3, rrs 4, and rps E showed that there was no base mutation in clinically sensitive and resistant strains to aminoglycosides. There were base mutations in rrs 3 and rrs4 of nine strains induced *in vitro*: there was a base mutation of A1409T in rrs 3 and rrs 4 of the highly resistant strain to GEN; there was a base mutation of A1408G in rrs3 and rrs4 of the highly resistant strain to KAN; and one highly resistant strain to SPE had a C1192T base mutation in rrs3 and rrs4, two strains had a C1192T base mutation in rrs3, and a sense mutation was not detected in the rpsE-encoding ribosomal protein S5. The strains were disrupted with the binding between aminoglycosides and the site in 16S rRNA due to inhibition of polypeptide synthesis (16). Base mutation and the corresponding amino acid mutation have been associated with aminoglycoside resistance. Thus, we concluded that the single base mutation of 16Sr RNA (rrs3 or rrs4) would mediate the resistance of *M. bovis* from yaks to aminoglycosides. The result was the same as that of previous studies and further confirmed the drug-resistance mechanism of *M. bovis* to aminoglycosides (16, 17). The previous research showed that all strains with high resistance to SPE harbored a single mutation the at rrs gene (C1192A in naturally resistant mutants and C1192T mutation in resistant mutants) (26). Meanwhile, there were A1408G and G1488A mutations in 16S rRNA of the Mb218 strain to GEN (27). This was similar to our study.

In our study, a single-site base mutation was detected in gyrA (Ser83Phe) or parC (Ser80Ile) for the clinically resistant strain, both base mutations were found in gyrA (Gly81Cys, Ser83Phe, and Glu87Asp or Lys) and parC (Ser80Ile, Ser81Tyr, and Asp84Asn or Tyr) in the highly resistant strain to fluoroquinolones. DNA gyrase and DNA topoisomerase IV were essential enzymes to fluoroquinolone targeting with two subunits (DNA gyrase: gyrA2gyrB2; DNA topoisomerase IV: parC2parE2). The base mutation in gyrA and parC was the main reason for the resistance to fluoroquinolone compared with that in gyrB and parE (19). The increasing fluoroquinolone resistance was found with both mutations in DNA gyrase and DNA topoisomerase IV (28). For *M. bovis*, the previous research showed that Ser83Phe in gyrA and Asp84Asn in parC point mutation were required for resistance to ENR (18). Japanese researchers found that resistant isolates to fluoroquinolones had a mutation in GyrA (Ser83Leu or Phe) and ParC (Ser81Pro or Ile) (19). The same results were also proved by a French researcher (29).

On the other hand, the previous research showed that the cytoplasmic drug concentrations were decreased by active efflux and reductions in influx. Plasmids were the type of DNA in bacteria and disseminated between cells. Plasmids could help bacteria cope with unfavorable environments by transfer accessory genes (30). The active efflux system results of *M. bovis* showed that there was no active efflux system based on fluoroquinolones and aminoglycosides expressed in *M. bovis* from yaks, and the existence of endogenous plasmids needs further study.

## Conclusion

The isolated strains of *M. bovis* from yaks in Tibet had varying degrees of resistance to fluoroquinolones and aminoglycosides. The mechanism of resistance to fluoroquinolones and aminoglycosides was identified preliminarily for *M. bovis* from yaks: the single-site base mutation mediated the resistance of *M. bovis* from yaks and both base mutations led to the highly resistant strain (aminoglycosides: rrs3 and rrs4; fluoroquinolones: gyrA and parC). The research could provide a reference for clinical treatment of *Mycoplasma bovis*.

## Data Availability Statement

The original contributions presented in the study are included in the article/supplementary material, further inquiries can be directed to the corresponding authors.

## Author Contributions

JN and SS conceived and designed the study. JN and MY executed the experiment and analyzed the sera and tissue samples. ZC, JX, and YX analyzed the data. All authors interpreted the data, critically revised the manuscript for important intellectual content, and approved the final version.

## Funding

This study was supported by the key research and development program of Tibet Autonomous Region (XZ202001ZY0046N) and the Chinese Agricultural Research Service (CARS-37).

## Conflict of Interest

The authors declare that the research was conducted in the absence of any commercial or financial relationships that could be construed as a potential conflict of interest.

## Publisher's Note

All claims expressed in this article are solely those of the authors and do not necessarily represent those of their affiliated organizations, or those of the publisher, the editors and the reviewers. Any product that may be evaluated in this article, or claim that may be made by its manufacturer, is not guaranteed or endorsed by the publisher.
